# Supplementary material for: Cryptic terrestrial fungus-like fossils of the early Ediacaran Period
Source: Nat Commun. 2021 Jan 28;12:641. doi: 10.1038/s41467-021-20975-1 (PMC7843733; doi:10.1038/s41467-021-20975-1)
Supplement: Supplementary file 1 — Supplementary Information [file 41467_2021_20975_MOESM1_ESM.pdf]

Supplementary Information for

Cryptic terrestrial fungus-like fossils of the early Ediacaran Period

Tian Gan, Taiyi Luo, Ke Pang, Chuanming Zhou, Guanghong Zhou, Bin Wan, Gang Li, Qiru Yi, Andrew D. Czaja, Shuhai Xiao

This file includes:

Supplementary Note 1: Stratigraphy and age constraints

Supplementary Note 2: Sheet-cavity cements

Supplementary Note 3: U-Pb radiometric dating of isopachous dolomite (ID)

Supplementary Note 4: Taphonomic discussion

Supplementary Figures

Supplementary Tables

Supplementary References

Supplementary Note 1: Stratigraphy and age constraints

Neoproterozoic sequence in the Weng'an area is exposed in a northeast-southwest trending anticline that is cut by several faults and divided into the northern Baiyan anticline and the southern Gaoping anticline[1] (Fig. 1a and b). The Datang and Beidoushan sections are located in the Baiyan anticline (Fig. 1b). The Ediacaran Doushantuo Formation at these two sections is ~30–40 m thick. It sits directly on glacial diamictite of the Cryogenian Nantuo Formation and underlies carbonates of the terminal Ediacaran Dengying Formation[2,3] (Fig. 1c, d). The Doushantuo Formation has been divided into five units: D1 (~1–4 m thick cap dolostone), D2 (~3–4 m thick siltstone and ~8–9 m thick lower phosphorite bed or Ore bed A), D3 (~2–4 m thick middle dolostone), D4 (~8–10 m thick upper phosphorite bed or Ore bed B), and D5 (~10 m thick phosphatic dolostone)[4-6] (Fig. 1c, d; Supplementary Fig. 1a). The Weng'an biota occurs in the D4 and D5 members. Unit D1 is the cap dolostone and it is capped by a karstic surface (Fig. 1c, d; Supplementary Fig. 1a, e, f). The cap dolostone is a distinctive sedimentary unit that is broadly distributed in South China and elsewhere in the world. The cap dolostone is characterized by tepee-like structures (interpreted as giant wave ripples[7]) and sheet-cracks[8]. The origin of the sheet-cracks is a matter of debated, and may be related to physical processes such as an increase in pore fluid pressure[8] or a decrease in hydrostatic pressure[9]. However, pre-existing sheet-cracks could be subsequently augmented by karstic dissolution (see below) to become sheet-cavities. Breccias and sheet-cavities occur at the base of the cap dolostone in an open mining pit at the Datang section (Supplementary Fig. 1b–d, g) and a mining tunnel at the Beidoushan section (Supplementary Fig. 1h). The sheet-cavities are typically filled with, from the margin to the center, isopachous dolomite (ID), fibrous calcite (FC), radial chalcedony (RC) or microcrystalline quartz (MQ), coarse quartz (CQ), and blocky calcite (BC) (Supplementary Fig. 4a, b; ref.[10]). A distinct karstic surface is observed at the top of the cap dolostone (Supplementary Fig. 1e, f), which has been previously recognized elsewhere in South China[11]. Locally the sheet-cavities with chalcedony cement are seen to be physically connected with the karstic surface atop the cap dolostone (Supplementary Fig. 1e). The age of the Doushantuo cap dolostone (D1)

in South China is well constrained to be ca. 635 Ma by a U-Pb zircon age of 634.6 ± 0.9 Ma from the topmost Nantuo Formation[12] and a U-Pb zircon age of 635.2 ± 0.6 Ma from the top of cap dolostone in South China[13]. In the Weng'an area, a whole-rock Pb–Pb isochron age of 599.3 ± 4.2 Ma was reported from the D4 Member at Weng'an[14]. In the Zhangcunping area north of the Yangtze Gorges, ash beds from strata equivalent to basal D3 and lower D4 units yielded U-Pb zircon ages of 614.0 ± 7.6 Ma[15] and 609 ± 5 Ma[16], respectively.

Supplementary Note 2: Sheet-cavity cements

In general, sheet-cavities in Ediacaran Doushantuo cap dolostone are centripetally filled with several different fabrics representing several generations of cementation and hydrothermal replacement (Supplementary Figs. 1; 4a, b): 1) isopachous dolomite (ID); 2) fibrous calcite botryoids (FC); 3) replacement of FC by hydrothermal radial chalcedony (RC) and quartz phases (MQ and CQ); and 4) calcareous paragenesis of blocky calcite (BC) ± dolomite ± barite (modified from refs.[10,11]). The occurrence of fabrics appears to be related to the dimension of sheet-cavities: smaller sheet-cavities are only filled with ID whereas larger ones filled with a complete cement sequence from ID to BC (Supplementary Fig. 4).

Isopachous dolomite (ID) occurs in both platform and slope facies in South China (e.g., Xiaofenghe, Jiulongwan, Weng'an, Daping, Wenghui, and Tianping sections) (Supplementary Fig. 4a–d and g–i) and elsewhere in the world (e.g., Namibia[9] and Australia[17]). ID is distinguished from dolomicrite in the cap dolostone by coarse euhedral dolospar (ca. 500 μm long) with crystal long-axis oriented perpendicularly to sheet-cavity walls. The basal part of ID is characterized by fine-grained radial or granular dolomite crystals, and the distal part typically consists of coarser rhombic dolomite. Zhou et al. (2010) proposed that ID cement in sheet-cavities was formed during a second transgression of regional or global scale after the Marinoan deglaciation[11]. Zhao et al. (2018), on the basis of rare earth element geochemistry, suggested that ID precipitated from the mixture of contemporaneous seawater and pore water near the water-sediment interface[18]. Considering the evidence for karstification related to post-glacial rebound and the termination of ID consistently followed by karstic FC, we infer that physically formed sheet-cracks may have been augmented by karstic dissolution in the phreatic zone and that ID precipitation probably occurred also in the phreatic zone by comparison with the vertical zoning of modern karst[19,20] (Supplementary Fig. 2c). A LA-ICP-MS U-Pb age of 632 ± 17 Ma was obtained from ID at the Daping section (see below; Supplementary Fig. 8; Supplementary Table 2), consistent with ID precipitation shortly after the termination of the Marinoan

glaciation[11].

Fibrous calcite botryoids (FC) is characterized by fine concentric laminae overlying ID and forming botryoidal structures. Partially silicified FC fabrics occur at the Daping and Xiaofenghe sections, where calcite laminae alternate with silica laminae and individual calcite laminae transition laterally into silica laminae (Supplementary Fig. 4b). Silicified botryoidal, stalagmite-like and stalactite-like structures occur in sheet-cavities at multiple sections in South China (Wenghui, Xiaofenghe, Beidoushan, Datang sections), and relics of FC have been identified at the Daping, Xiaofenghe and Wenghui sections[21] (Supplementary Figs. 2a, b; 4b). Thus, these structures are interpreted as silicified paleo-speleothems. At the Datang and Beidoushan sections in the Weng'an area, filamentous fossils are mainly preserved in silicified structures with a core of microcrystalline quartz (MQ) and a shell of organic-rich, laminated, botryoidal radial chalcedony (RC). This core-shell structure is similar to modern moonmilk from the Grotta Cesare Battisti cave, North Italy[22]. The microcrystalline core may be the result of microbially mediated nucleation and mineralization[23,24] and the external clay-rich or organic-rich laminated shell may represent subsequent, relatively slow precipitation[25,26]. Considering the widespread karstification in the cap dolostone in South China following post-glacial rebound, we propose that CO_2 degassing in the vadose zone drove the precipitation of paleo-speleothems characterized by FC (Supplementary Fig. 2d). The formation of sheet-cavities and precipitation of FC are probably related to the karstification surface at the top of the cap dolostone (Supplementary Fig. 1e–f), which has been constrained by an ash bed of ~635.2 Ma at the top of the cap dolostone[13].

Radial chalcedony (RC), microcrystalline quartz (MQ), and coarse quartz (CQ) occur in sheet-cavities in most of the sections in South China, and they are interpreted as replacement fabrics related to hydrothermal activities (Supplementary Figs. 1e–f, 2e). RC with or without lamellar pyrite forms isopachous layers (0.5–2.0 mm thick) overlying ID. At the Xiaofenghe and Wenghui sections, partially silicified RC can be observed, with RC and FC vertically alternating with each other or laterally transitioning into each other (Supplementary Fig. 4b). MQ fabrics typically form the

core of RC fabrics and consist of crystals 30–80 μm in diameter, whereas CQ fabrics lie outside the RC botryoids and consist of crystals 200–900 μm in diameter (Supplementary Fig. 4d), suggesting gradually stable crystal growth toward the end of chalcedony formation. Fluid inclusions in CQ from the Jiulongwan section in the Yangtze Gorges area typical have homogenization temperatures of 160–220 °C (mean = 192 °C; n = 31) and two salinity peaks (6.3–8.3 wt.% NaCl equiv. and 18.0–20.8 wt.% NaCl equiv.), indicating a mixture of high- and low-salinity hydrothermal fluids[10]. Furthermore, Cui et al. (2019) proposed that fluids for silica precipitation in sheet-cavities originated from a mixture of contemporaneous seawater and hydrothermal fluids based on the low Ge/Si ratio, depleted LREE pattern, and positive Eu anomalies in the silica phase[27]. In addition, the karstic surface atop the cap dolostone at Datang shows decimeter-scale topographic reliefs (Supplementary Fig. 1e–f) and is sometimes also silicified (Supplementary Fig. 1e, where a chalcedony ring is exposed and possibly represents a hot spring system connecting the sheet-cavities and the karstic surface), indicating that the silicified fabrics were weathered together with the cap dolostone during the karstification event that occurred before 635.2 Ma (based on the age of an ash bed at the top of the cap dolostone[13]).

There are two phases of blocky calcite fabrics (BC): white blocky calcite (WBC) and dark blocky calcite (DBC), which fill the remaining space within sheet-cavities. White blocky calcite (WBC) is often accompanied with barite and dolomite. Zhou et al. (2017) inferred that WBC was the product of hydrothermal activity after silicification[10]. The geochemical characters of MREE enrichment, positive Eu anomalies, insignificant Ce anomalies, and high Y/Ho ratios indicate that WBC precipitated from a mixture of Marinoan deglacial seawater and hydrothermal fluids with a temperature of at least 105 °C as estimated by oxygen isotope[18]. It should be noted that WBC has higher $\delta^{13}C$ values (> –12‰)[8,18] than DBC. Dark blocky calcite (DBC) from the Jiulongwan, Huajipo, and Wangzishi sections in Hubei Province have extremely negative $\delta^{13}C$ values (< –30‰)[8,28-31], probably related to oxidation of methane hydrate[31] or thermogenic methane[32].

Based on the above discussion, the sequence of mineral precipitation and

replacement in sheet-cavities is summarized in Supplementary Figure 2, emphasizing the relationship between post-glacial rebound, karstification, and dissolution and precipitation in the phreatic and vadose zones. The fungus-like microfossils reported in this study are preserved mostly in the silicified botryoidal structures with microcrystalline quartz core and radial chalcedony fabrics (RC), and we infer that these fungus-like micro-organisms colonized the cryptic cavities during the karstification.

Supplementary Note 3: U-Pb radiometric dating of isopachous dolomite (ID)

Isopachous dolomite cement in Doushantuo sheet-cavities was chosen for *in-situ* radiometric dating. After optical examination and LA-ICP-MS elemental analyses, suitable domains with high U/Pb ratios and no fractures were targeted for isotopic analysis using LA-ICP-MS to provide the best achievable precision and accuracy. Uranium concentration of analyzed samples ranges from 0.81 to 9.66 ppm, total lead from 0.17 to 71.57 ppm, $^{238}U/^{206}Pb$ from 0.102 to 7.970, and $^{207}Pb/^{206}Pb$ from 0.237 to 0.853. A total of 13 spots were ablated on 13 dolomite crystals from two thin sections (Supplementary Table 2), yielding an intercept age of 632 ± 17 Ma (MSWD = 1.6) (Supplementary Fig. 8), which is interpreted as the age of isopachous dolomite cementation in Doushantuo sheet-cavities.

Supplementary Note 4: Taphonomic discussion

Cyanobacteria, bacteria, fungi, and algae have been found in modern speleothems[33-41], and culture experiments have shown that microbial activities can effectively promote the growth of carbonate minerals[42-44]. Speleothems are a suitable habitat for subaerial biofilms, and this is particularly true for certain speleothems such as moonmilk and flowstone, the formation of which are considered to be related to microbial activities[45]. Some microbial fossils, including bacterial and fungal fossils, have been known to be preserved in Jurassic calcareous crusts[46], and dendritic cyanobacteria fossils in early Cretaceous micro-karst[47]. Thus, in principle, it is possible for microfossils to be preserved in karstic precipitates.

The fossiliferous RC and MQ fabrics in sheet-cavities of the cap dolostone at the Datang and Beidoushan sections are interpreted as moonmilk-like coatings on fissure walls (Supplementary Fig. 4d–k). The rapid precipitation of paleo-speleothems in the sheet-cavities may have three-dimensionally encased the filamentous fungus-like micro-organisms in carbonate minerals (i.e., FC fabric), which were later replaced by hydrothermal chalcedony (RC or MQ fabric). Although many details of the taphonomic processes responsible for the preservation of the Doushantuo fungus-like fossils remain unknown, a possible sequence of events can be summarized as follow (Supplementary Fig. 2). (1) At the termination of the Marinoan glaciation at ~635 Ma, post-glacial rebound resulted in subaerial exposure of the Doushantuo cap dolostone, and pre-existing sheet-cracks[8,9] were augmented through local dissolution in CO_2-rich meteoric water to form sheet-cavities. (2) Carbonate precipitates, first the ID fabric and then the FC fabric, were precipitated in sheet-cavities. The ID fabric was precipitated near the phreatic zone, whereas the FC fabric in characteristic speleothems was likely formed when ground-water that was saturated with respect to $CaCO_3$ seeped in the vadose zone. A karst-microbial system including filamentous fungus-like micro-organisms was established in this stage. Fungus-like filaments and other micro-organisms were encased in speleothem deposits. (3) Subsequent events include pyritization of fungus-like micro-organisms, selective replacement of FC by RC, MQ, and CQ fabrics related to hydrothermal activities, and the precipitation of blocky calcite (BC). The relative

timing of these last three events is uncertain, although it is likely that pyritization probably predated RC/MQ/CQ replacement and that these events occurred before the deposition of unit D2, certainly before ca. 632 Ma.

Supplementary Figures

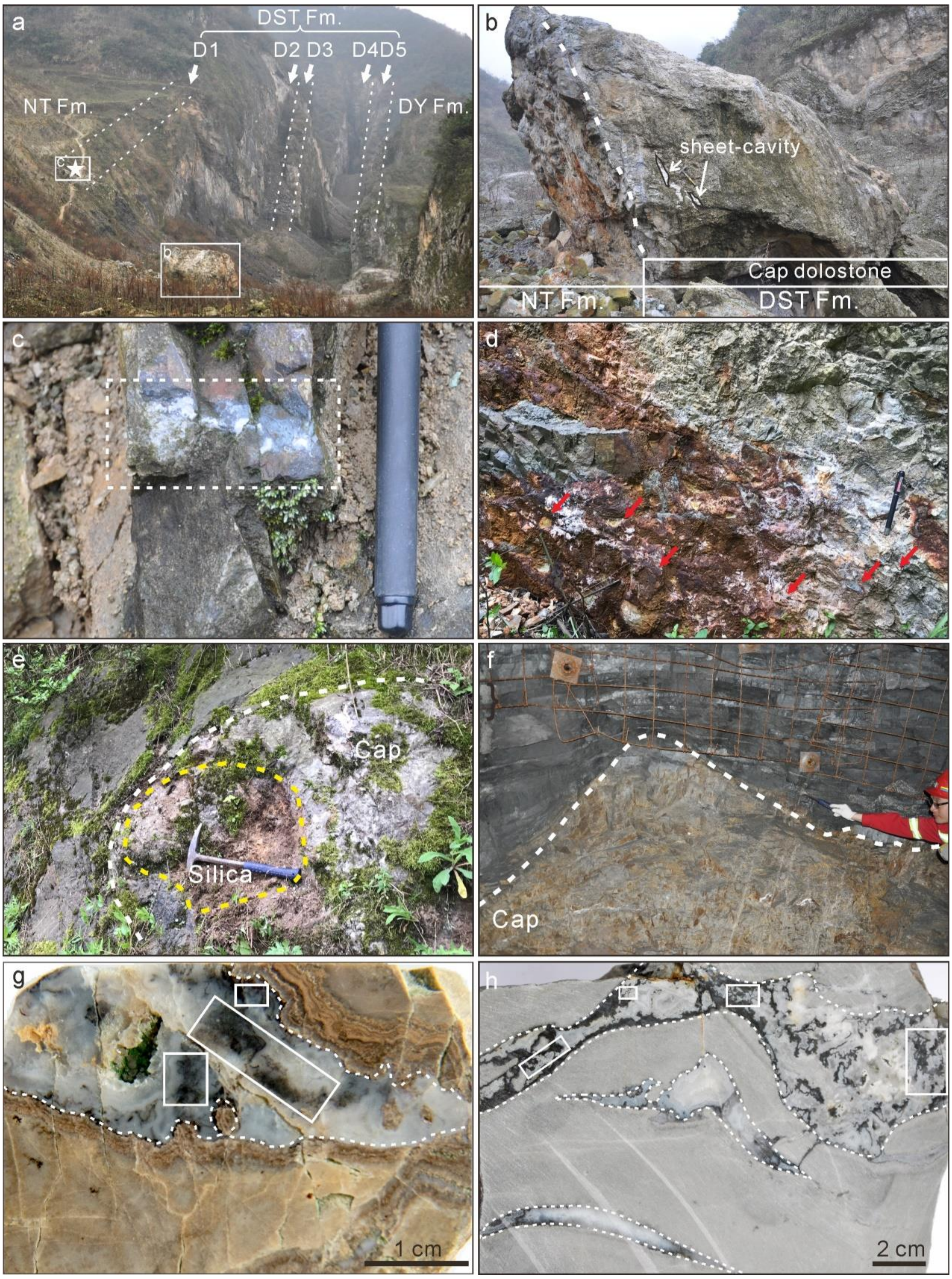

Supplementary Figure 1 | Stratigraphic sequence, sheet-cavities, paleo-karstic features, and polished slabs of cap dolostone. a, Stratigraphic sequence of Doushantuo Formation at Datang, with stratigraphic units marked (NT Fm. = Nantuo Formation; DST Fm. = Doushantuo Formation; DY Fm. = Dengying Formation). Labelled boxes mark areas magnified in **b** and **c**. Star marks fossil locality. **b**, A float

showing boundary (dashed line) between the Nantuo Formation and cap dolostone of the basal Doushantuo Formation. **c**, Sheet-cavity in cap dolostone. Box denotes fossiliferous cement infilling sheet-cavity. **d**, Basal cap dolostone at Datang, with arrows highlighting clasts in the cap dolostone. **e, f**, Karstic surface (white dashed lines) atop cap dolostone at Datang. Yellow dashed line denotes a silica ring (possible a hot spring channel and part of the sheet-cavity system). **g, h**, Polished slabs showing fossiliferous siliceous cement in sheet-cavities. Dashed lines highlight boundary between sheet-cavity cement and cap dolostone. Boxes denote fossiliferous siliceous cement in sheet-cavities. **g** is from Datang (sample 17DT-A-9) and **h** is from Beidoushan (sample 18BD-25). Hammers in **b** (between arrows) and **f** (held by geologist) are ~30 cm long, and hammer in **e** is ~40 cm long. Pens in **c** and **d** are ~1 cm in diameter.

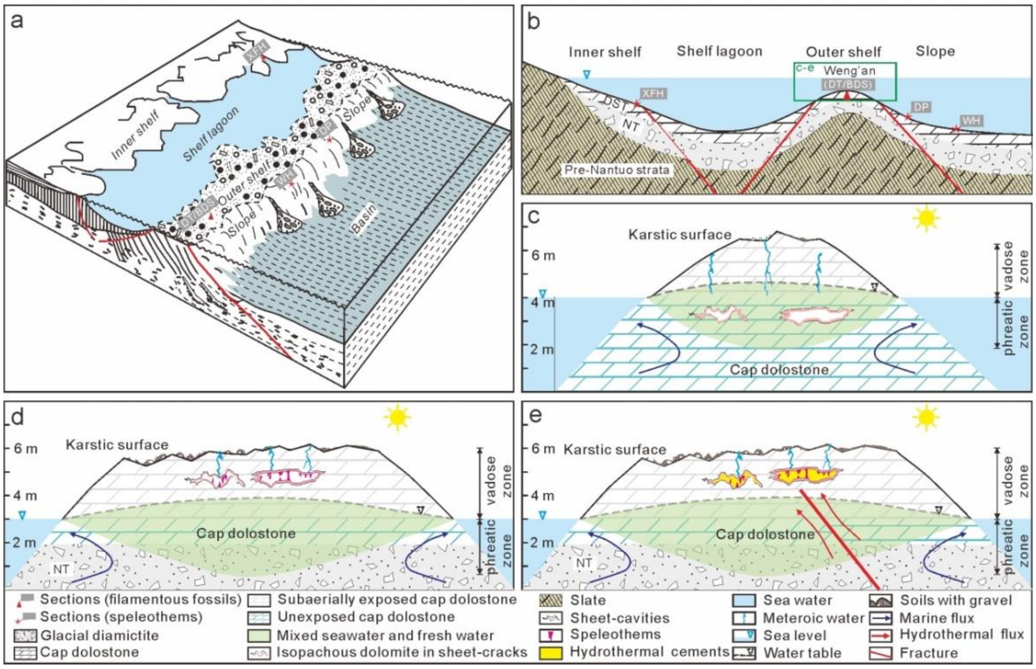

Supplementary Figure 2 | Paleogeographic reconstruction (a–b) and a model of sheet-cavity cement formation (c–e). **a**, Depositional environment of the Doushantuo Formation in South China. **b**, Cross section from inner shelf to slope facies, showing the Doushantuo cap dolostone (DST) overlying terminal Cryogenian glacial diamictite of the Nantuo Formation (NT). Red triangles and stars in (**a**–**b**) mark inferred paleo-environmental location of sites mentioned in the text: XFH – Xiaofenghe; DT – Datang; BDS – Beidoushan; DP – Daping; WH – Wenghui. Green rectangle in (**b**) marks area magnified in (**c**–**e**). **c**, Early stage of post-glacial rebound: sheet-cavities were formed or augmented in the phreatic zone through dissolution and lined with isopachous dolomite (ID). **d**, Later stage of post-glacial rebound: sheet-cavities were elevated to the vadose zone, where speleothems with fibrous calcite (FC) developed. **e**, Hydrothermal fluids were introduced, calcareous speleothems were replaced by radial chalcedony (RC), and hydrothermal cements, including microcrystalline quartz (MQ) and coarse quartz (CQ), were precipitated. **a**–**b**, modified from ref.[6]. **c**–**e**, based on modern mixing dolomitization model[19] and karst zone models[20,48]. Maximum distance between sheet-cavities and karstic surface atop the cap dolostone is 4 meters (i.e., the thickness of the cap dolostone), and sometimes sheet-cavities are exposed on and physically connected with the karstic surface (Supplementary Fig. 1e).

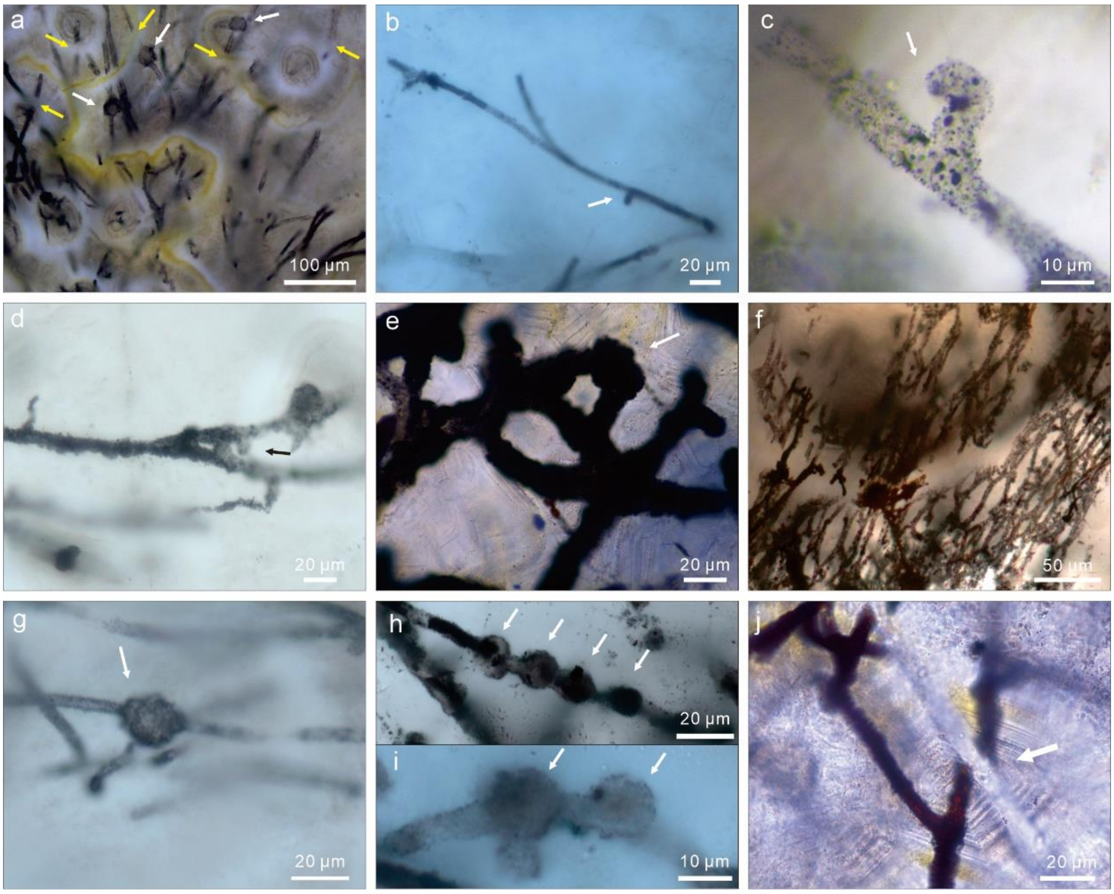

Supplementary Figure 3 | Type A filaments and associated small spheres. a, Aggregate of Type A filaments with small hollow spheres (white arrows). Filaments are embedded in but disrupted by chalcedony botryoids (yellow arrows). **b**, Multiple orders of branches and a short lateral branch (arrow). **c**, Short and bent lateral branch (arrow). **d**, Branching filaments with secondary lateral branches to form a ladder-like branching system (arrow). **e, f,** Networks of extensively fused filaments (arrow). **g,** Small intercalary sphere (arrow). **h**, **i**, Small concatenated spheres (arrows) coaxially aligned with filaments. **j**, Tpye A filaments cut and offset by late diagenetic veins (arrow). For each illustrated specimens in this Supplementary Figure, its repository information is given in Supplementary Table 3.

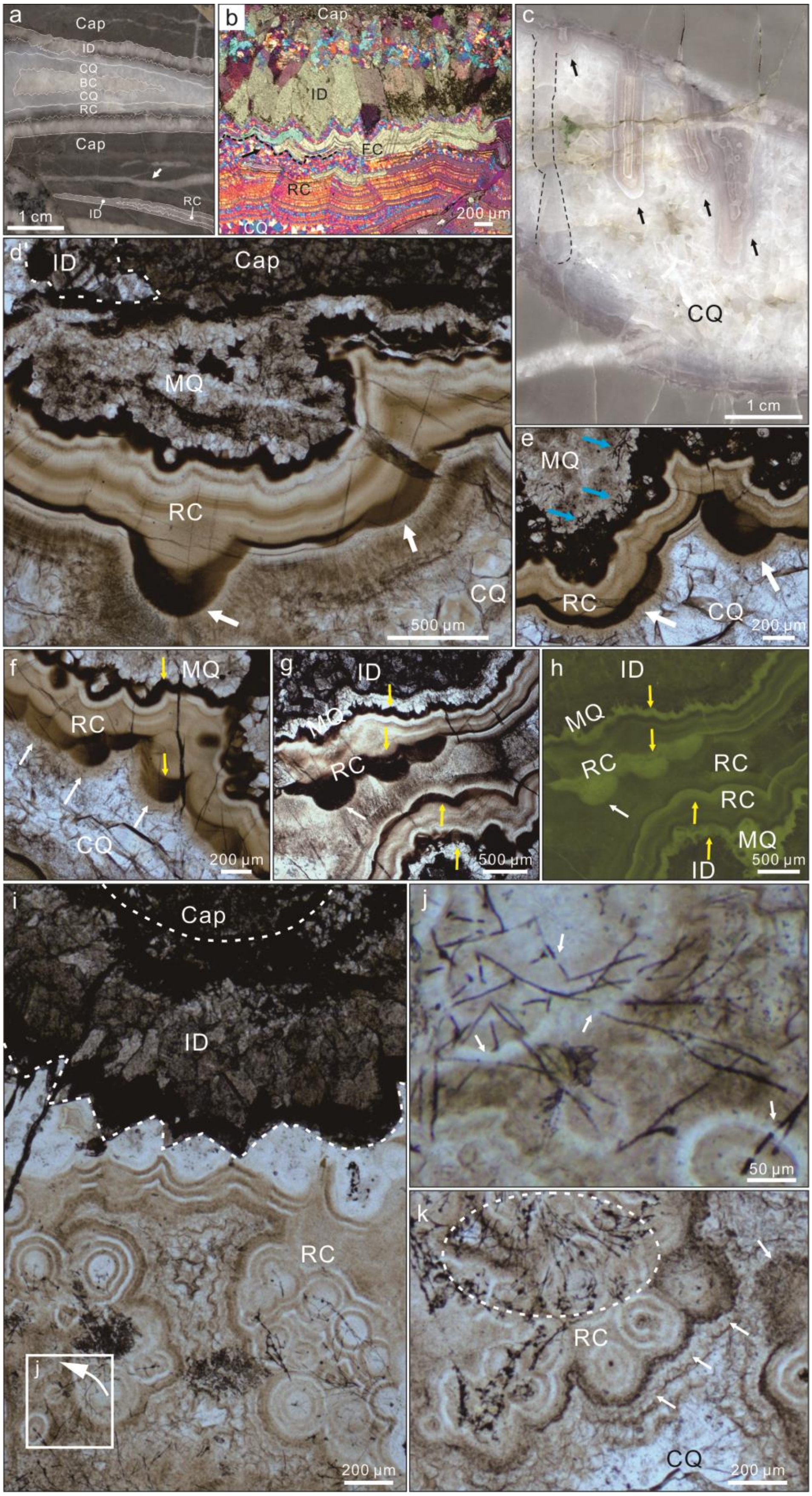

Supplementary Figure 4 | Petrographic thin sections showing different

cementation fabrics (ID, isopachous dolomite; FC, fibrous calcite botryoids; RC, radial chalcedony; MQ, microcrystalline quartz; CQ, coarse quartz; BC, block calcite) in sheet-cavities. a, b Polished slab (**a**) and thin section photomicrograph (under cross-polarized light with a gypsum plate) (**b**) showing relationship of cap dolostone (cap) with ID, FC, RC, CQ, and BC. Note in **a** that larger sheet-cavities can be filled with a complete cement sequence from ID to BC, whereas smaller ones are filled by ID only (white arrow). Note in **b** partial replacement of FC by RC (i.e., individual FC laminae, which have high-order white birefringence colors, abruptly transition laterally to RC laminae, which have orange-red-blue birefrigence colors, black arrow). **c**, Polished slab showing stalactites (black arrows) and a stalagnate (black dash line) in sheet-cavity from Beidoushan, adopted from ref.[21]. **d**, TLM photomicrograph showing relationship between cap dolostone (Cap) and cement fills in sheet-cavity. Only half of the sheet-cavity is shown and cementation progressed from top to bottom, including ID, MQ, RC, and CQ fabrics. MQ probably replaced originally crystalline calcite farbics and contains poorly preserved filaments. RC probably replaced originally fibrous calcite fabrics. Dashed lines mark ID. White arrows denote siliceous botryoids. **e**, TLM photomicrograph showing a core of MQ, RC botryoids (white arrows), and CQ fabrics. Filamentous microfossils (blue arrows) were mostly preserved in the core (MQ) of the core-shell structure. **f–h**, Botryoidal structures (RC), MQ, and CQ under TLM (**f** and **g**) and ELM (**h**, same area as **g**). White arrows denote botryoidal structures and yellow arrows denote organic-rich zones. **i**, TLM photomicrograph of botryoidal structures (RC) that host filamentous microfossils. Dashed lines bound isopachous dolomite (ID). **j**, Enlargement of labeled rectangle in **i** (with a 90° counterclockwise rotation), showing fossil filaments. Note that filaments are disrupted by botryoidal bands (arrows). **k**, Siliceous botryoids (RC), CQ, and aggregate of fossil filaments (dashed line). Arrows denote botryoids. **a** is from Daping in Hunan province (sample 19DP2-2l); **b** is from Xiaofenghe in Yangtze Gorges area (sample XF2-5); **c** (sample 18BDS-2), **d, f** (sample 18BD-6), **e** (sample 18BD-12), and **g–h** (sample 18BD-3) are from Beidoushan in Guizhou province; and **i–k** (sample 17DT-A-9) are from Datang in Guizhou province.

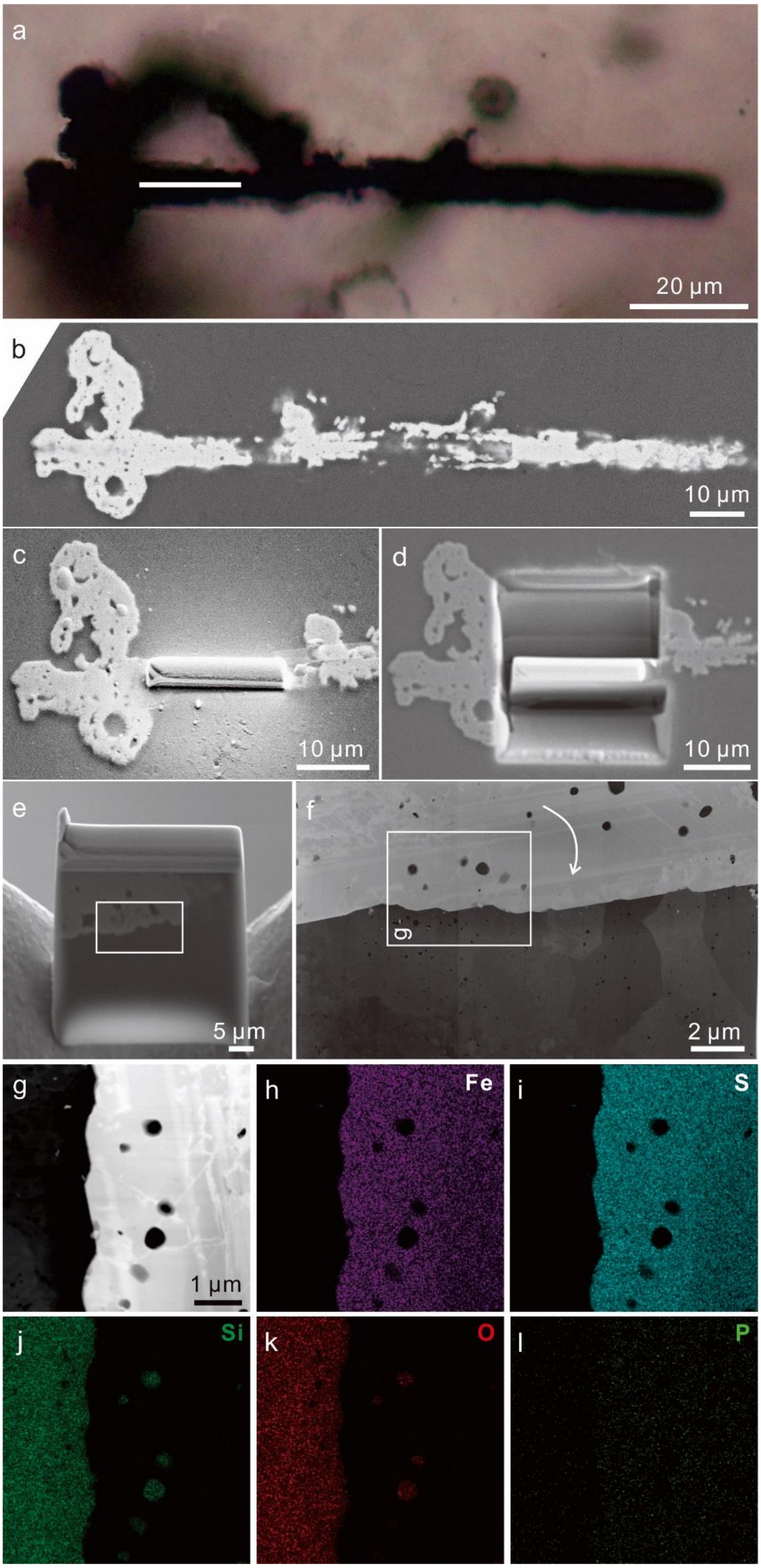

Supplementary Figure 5 | TLM, FIB-SEM, and STEM of Type A filament. a,

TLM photomicrograph of Type A filament (opaque) in silica matrix (transluscent), with white line denoting position of FIB cut. **b,** Back scattered electron SEM micrograph of specimen in **a**. **c**, Secondary electron (SE) SEM micrograph of left part of specimen in **b** before FIB cut. **d**, SE SEM micrograph after initial FIB step-cut. **e**, SE SEM micrograph after FIB step-cut and thinning. **f**, STEM overview of ultrathin foil corresponding to rectangle in **e**. **g**, Magnification of rectangle in **f** (with a 90 ° clockwise rotation). The bright field represents the filament and the dark field represents the matrix in **e–g**. **h–l**, EDS elemental maps of **g**, with elements marked in upper right. The filament is enriched in iron and sulfur whereas the matrix is enriched in silicon and oxygen, indicating that the filament is pyritized. Analyzed specimen is from Datang (sample 16DT-2).

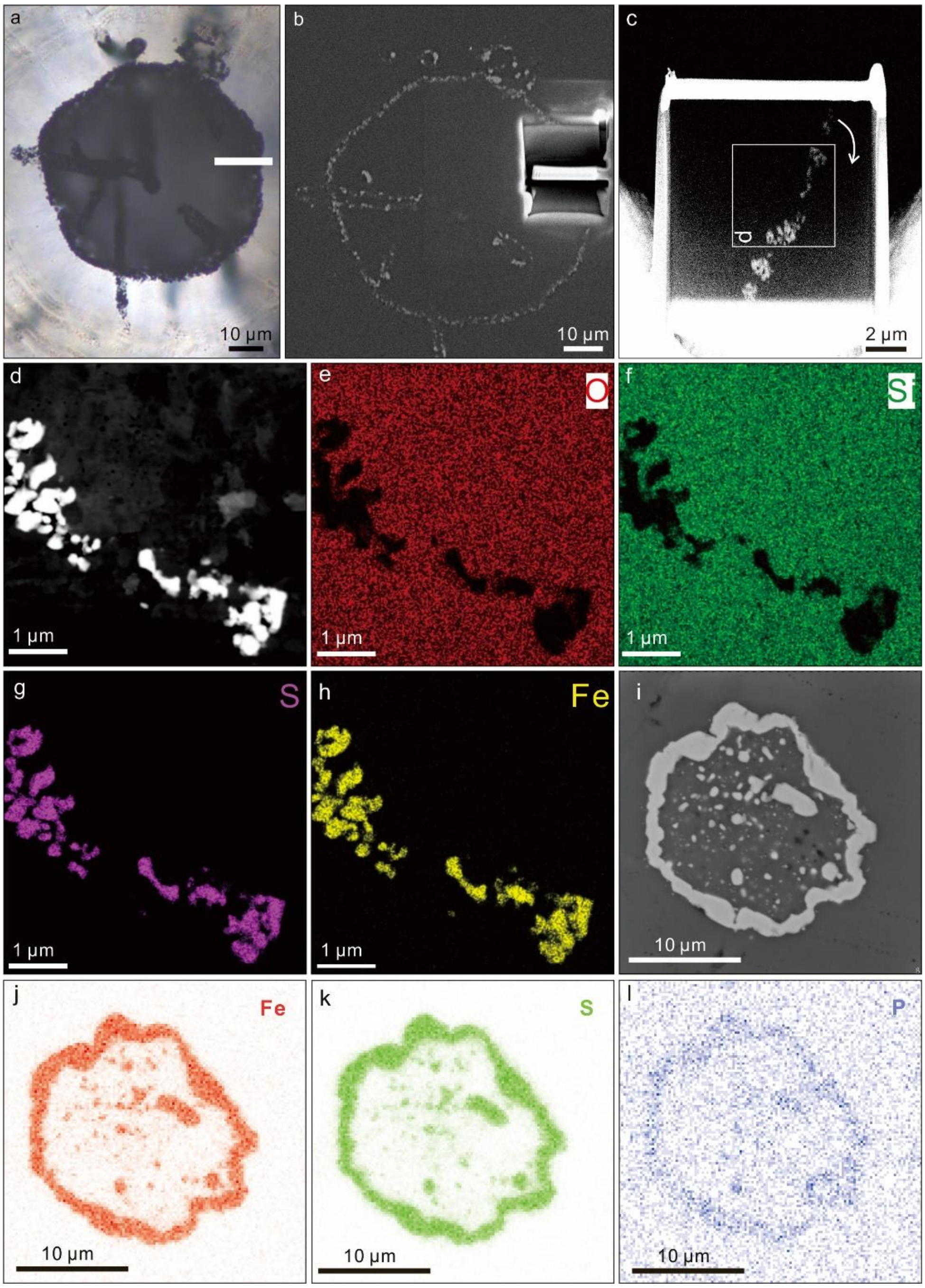

Supplementary Figure 6 | TLM, SEM, FIB-SEM, STEM, and elemental maps of a large sphere (a–h) and a small sphere (i–l). a, TLM photomicrograph of a large sphere in cross section (opaque circular rim). The sphere is filled and surrounded by transluscent silica, and it is penetrated by several Type A filaments (opaque elongate structures). White line denotes position of FIB cut. **b**, Secondary electron (SE) SEM micrograph of specimen in **a** after initial FIB step-cut. The bright field represents the pyritized sphere and filaments in cross section, whereas the dark field represents the silica matrix. **c**, SE SEM micrograph after FIB step-cut and thinning. **d**, STEM micrograph of ultrathin

foil corresponding to rectangle in **c** (with a 90 ° clockwise rotation). **e–h**, EDS elemental maps of **g**, with elements marked in upper right. The wall of the large sphere is enriched in iron and sulfur and the matrix is enriched in silicon and oxygen, indicating that the wall of the large sphere is pyritized. **i**. Back scattered electron SEM micrograph of a small sphere in cross section. The bright ring represents the wall of the small sphere and the surrounding dark field represents the silica matrix. **j–l**, EDS elemental maps of **i**, with elements marked in upper right. The wall of the small sphere is enriched in iron and sulfur and weakly elevated in phosphorus, indicating that it is pyritized. Specimens are from Datang (**a–h** from sample 17DT-A-9a; **i–l** from sample 16DT-2).

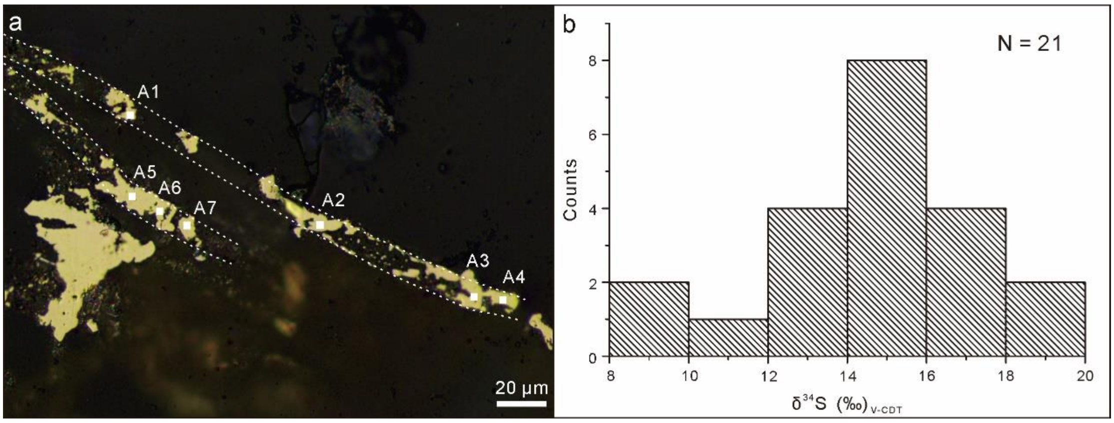

Supplementary Figure 7 | Nano-SIMS pyrite sulfur isotope values of Type A filaments. a, RLM photomicrograph of Type A filaments. Dotted lines mark filaments and white squares mark areas chosen for Nano-SIMS analysis. Two to four duplicate analyses were measured in each area. **b**, Frequency distribution of $\delta^{34}S_{pyrite}$ values. Source data for sulfur isotope values are provided in Supplementary Table 1. Analyzed specimens are from Datang (sample 16DTC-2A).

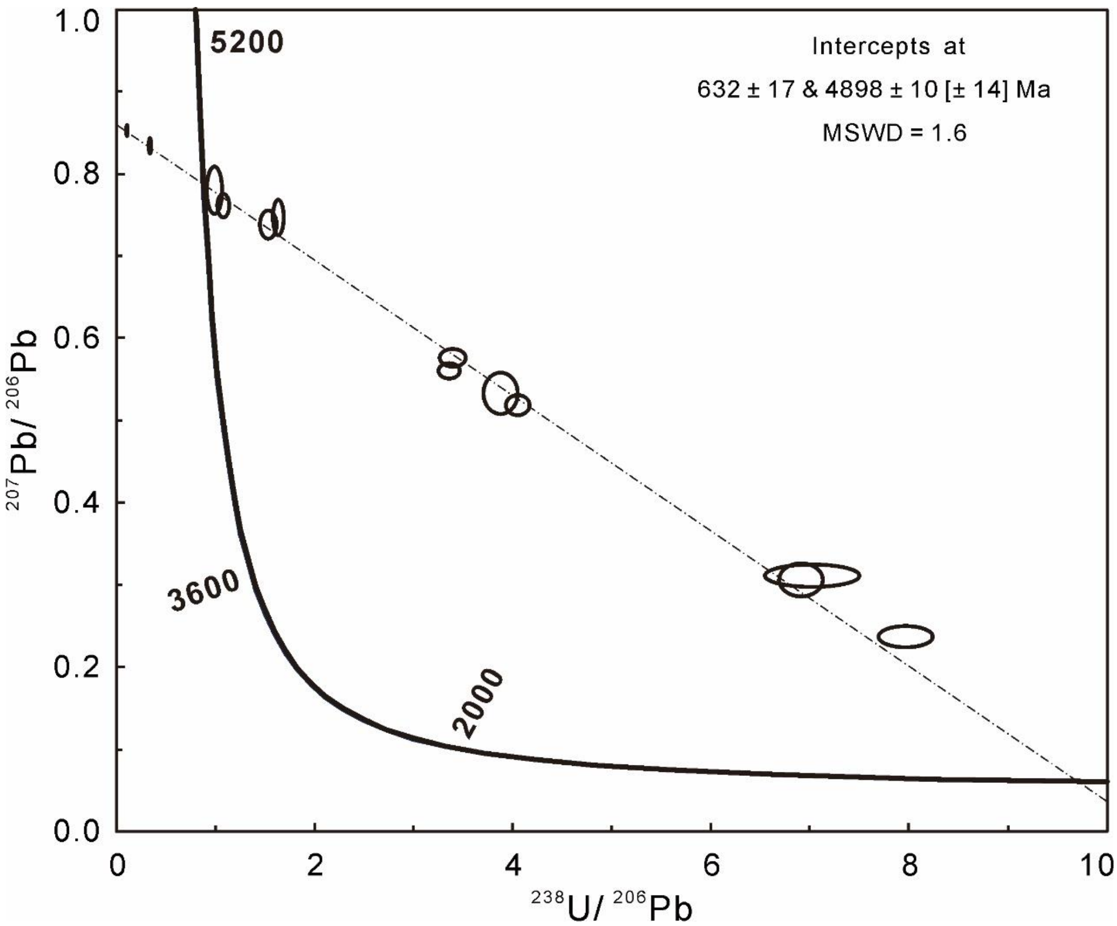

Supplementary Figure 8 | U-Pb discordia diagram for isopachous dolomite samples dated via LA-ICP-MS. Error ellipses are 1σ. Source data for LA-ICP-MS U-Pb dating are provided in Supplementary Table 2

Supplementary Tables

Supplementary Table 1 | Nano-SIMS pyrite $\delta^{34}S$ data of Type A filaments. Analyzed areas (A1–A7) and frequency distribution of $\delta^{34}S$ data are shown in Supplementary Figure 7. Sulfur isotope measurements were calibrated using the Sonora pyrite standard[49,50].

Sample	$\delta^{34}S_{V\text{-}CDT}$ (‰)	± SE (‰)	Analyzed area and spot
16dt-2-s1_mg_1	15.4	0.2	A1; S1-1
16dt-2-s1_mg_2	14.7	0.2	A1; S1-2
16dt-2-s1_mg_3	15.1	0.2	A1; S1-3
16dt-2-s2_mg_1	16.0	0.2	A2; S2-1
16dt-2-s2_mg_2	15.5	0.2	A2; S2-2
16dt-2-s2_mg_3	14.6	0.2	A2; S2-3
16dt-2-s3_mg_1	14.9	0.2	A3; S3-1
16dt-2-s3_mg_2	13.8	0.2	A3; S3-2
16dt-2-s3_mg_3	13.7	0.2	A3; S3-3
16dt-2-s4_mg_1	16.2	0.2	A4; S4-1
16dt-2-s4_mg_2	10.4	0.2	A4; S4-2
16dt-2-s4_mg_3	12.4	0.2	A4; S4-3
16dt-2-s5_mg_1	18.2	0.2	A5; S5-1
16dt-2-s5_mg_2	9.2	0.2	A5; S5-2
16dt-2-s6_mg_1	9.3	0.2	A6; S6-1
16dt-2-s6_mg_2	17.7	0.2	A6; S6-2
16dt-2-s6_mg_3	15.0	0.2	A6; S6-3
16dt-2-s7_mg_1	16.4	0.2	A7; S7-1
16dt-2-s7_mg_2	16.4	0.2	A7; S7-2
16dt-2-s7_mg_3	13.1	0.2	A7; S7-3
16dt-2-s7_mg_4	18.7	0.2	A7; S7-4

Supplementary Table 2 | LA-ICP-MS U-Pb data of isopachous dolomite (ID).

Spot	Sample	$^{Total}Pb^{§}$ ppm	$^{232}Th^{§}$ ppm	$^{238}U^{§}$ ppm	$^{238}U/^{206}Pb^{*}$	±1σ (%)	$^{207}Pb/^{206}Pb^{\#}$	±1σ (%)	rho
A13	14DP-1C1-9	71.57	0.02	2.13	0.102	2.3	0.853	0.5	0.05
A14	19DPc1-3-17	8.66	0.04	0.81	0.337	2.0	0.834	0.7	0.05
A15	14DP-1C1-10	13.40	0.01	4.40	0.992	5.4	0.780	2.4	0.12
A16	14DP-1c1-32	6.18	0.00	2.06	1.079	3.9	0.762	1.3	0.10
A17	19DPc1-3-19	1.85	0.00	0.87	1.530	3.6	0.738	1.6	0.09
A18	14DP-1C1-15	7.60	0.00	4.09	1.637	2.1	0.746	1.9	0.05
A19	14DP-1C1-22	4.53	0.01	6.06	3.356	2.1	0.561	1.1	0.05
A20	14DP-1C1-23	1.86	0.00	2.18	3.393	2.5	0.576	1.3	0.06
A21	14DP-1C1-26	6.27	0.03	9.66	3.880	3.0	0.533	3.2	0.07
A22	14DP-1c1-29	1.51	0.00	2.42	4.052	1.9	0.518	1.5	0.05
A23	14DP-1C1-17	0.67	0.00	2.38	6.913	2.1	0.306	4.3	0.05
A24	14DP-1C1-16	1.58	0.00	5.11	7.027	4.4	0.311	3.0	0.10
A25	19DPc1-3-2	0.17	0.00	0.85	7.970	2.2	0.237	3.7	0.06

Note: $^{Total}Pb^{§}$, $^{232}Th^{§}$ and $^{238}U^{§}$ contents of samples were calculated relative to NIST 614.

$^{238}U/^{206}Pb^{*}$ fractionations of samples were normalized to reference WC-1.

$^{207}Pb/^{206}Pb^{\#}$ fractionations of samples were normalized to NIST 614.

rho is the error correlation coefficient defined as the quotient of the propagated errors of the $^{206}Pb/^{238}U$ and $^{207}Pb/^{235}U$ ratios.

Supplementary Table 3 | List of illustrated specimens.

Figure number	NIGPAS Museum catalogue number	Thin section number	Sample number	Location	Coordinates (England Finder)
Fig. 2a	PB21760	18DT-A-9b	17DT-A-9	Datang	G21/2, G22
Fig. 2b	PB21761	18DT-A-9a	17DT-A-9	Datang	G6/3
Fig. 2c	PB21762	17DT-A-9a	17DT-A-9	Datang	S9/3
Fig. 2d	PB21760	18DT-A-9b	17DT-A-9	Datang	J23/4
Fig. 2e	PB21761	18DT-A-9a	17DT-A-9	Datang	G8/3
Fig. 2f	PB21761	18DT-A-9a	17DT-A-9	Datang	Q36/3
Fig. 2g–i	PB21760	18DT-A-9b	17DT-A-9	Datang	G21/2
Fig. 2j	PB21763	18DT-A-9c	17DT-A-9	Datang	U19/2, U19/4, U20/1, U20/3
Fig. 2k	PB21764	17DT-A-9b	17DT-A-9	Datang	U22/1, U22/3
Fig. 3a	PB21765	17DT-A-9-1	17DT-A-9	Datang	J28, J29, K28, K29
Fig. 3b	PB21766	17DT-A-6	17DT-A-6	Datang	O19/4
Fig. 3c	PB21764	17DT-A-9b	17DT-A-9	Datang	S10/4
Fig. 3d	PB21764	17DT-A-9b	17DT-A-9	Datang	T10/1
Fig. 3e	PB21767	18BD-33	18BD-33	Beidoushan	X7/2
Fig. 3f	PB21764	17DT-A-9b	17DT-A-9	Datang	U9/2
Fig. 3g	PB21768	18BD-35	18BD-35	Beidoushan	T34/2
Fig. 4a	N/A	18DT-A-9g	17DT-A-9	Datang	N/A
Fig. 4b	PB21761	18DT-A-9a	17DT-A-9	Datang	G6
Fig. 4c	N/A	18DT-A-9g	17DT-A-9	Datang	N/A
Fig. 4d	PB21762	17DT-A-9a	17DT-A-9	Datang	X30/4, X31/3, Y30/2, Y30/1
Fig. 4e	PB21762	17DT-A-9a	17DT-A-9	Datang	X28/3
Fig. 4f	PB21762	17DT-A-9a	17DT-A-9	Datang	B22/4
Fig. 4g–k	PB21769	17DT-A-9-1mm	17DT-A-9	Datang	From a 1 cm × 1 mm thin section
Supplementary Fig. 3a	PB21764	17DT-A-9b	17DT-A-9	Datang	D29
Supplementary Fig. 3b	PB21762	17DT-A-9a	17DT-A-9	Datang	P11/4, P12/3
Supplementary Fig. 3c	PB21770	18BD-25a	18BD-25	Beidoushan	J7/3
Supplementary Fig. 3d	PB21771	18DT-A-9d	17DT-A-9	Datang	N13/1
Supplementary Fig. 3e	N/A	18DT-A-9g	17DT-A-9	Datang	N/A
Supplementary Fig. 3f	PB21762	17DT-A-9a	17DT-A-9	Datang	S10/2
Supplementary Fig. 3g	PB21761	18DT-A-9a	17DT-A-9	Datang	F9/3
Supplementary Fig. 3h	PB21772	18BD-6	18BD-6	Beidoushan	J11/3, K11/1

Supplementary Fig. 3i	PB21772	18BD-6	18BD-6	Beidoushan	O15/1
Supplementary Fig. 3j	PB21762	17DT-A-9a	17DT-A-9	Datang	H13/3

Note: Thin section 18DT-A-9g was abraded because of sample preparation for SRXTM; All the other specimens illustrated in this paper are reposited in Nanjing Institute of Geology and Palaeontology (NIGPAS, Nanjing, China), with a NIGPAS museum catalog number (prefix PB-) given for each thin section.

Supplementary References

1 Zhang, Y. & Yuan, X. New data on multicellular thallophytes and fragments of cellular tissues from late Proterozoic phosphate rocks, South China. *Lethaia* **25**, 1–18 (1992).

2 Zhao, Z., Xing, Y., Ma, G. & Chen, Y. *Biostratigraphy of the Yangtze Gorge Area, (1) Sinian*. (Geological Publishing House, 1985).

3 Zhao, Z. *et al. The Sinian System of Hubei*. (China University of Geosciences Press, 1988).

4 Chen, D. F., Dong, W. Q., Zhu, B. Q. & Chen, X. P. Pb-Pb ages of Neoproterozoic Doushantuo phosphorites in South China: constraints on early metazoan evolution and glaciation events. *Precambrian Res.* **132**, 123–132 (2004).

5 Xiao, S., Zhang, Y. & Knoll, A. H. Three-dimensional preservation of algae and animal embryos in a Neoproterozoic phosphorite. *Nature* **391**, 553 – 558 (1998).

6 Xiao, S. *et al.* The Weng'an biota and the Ediacaran radiation of multicellular eukaryotes. *Natl. Sci. Rev.* **1**, 498–520 (2014).

7 Allen, P. A. & Hoffman, P. F. Extreme winds and waves in the aftermath of a Neoproterozoic glaciation. *Nature* **433**, 123–127 (2005).

8 Jiang, G., Kennedy, M. J., Christie-Blick, N., Wu, H. & Zhang, S. Stratigraphy, sedimentary structures, and textures of the late Neoproterozoic Doushantuo cap carbonate in South China. *J. Sediment. Res.* **76**, 978–995 (2006).

9 Hoffman, P. F. & Macdonald, F. A. Sheet-crack cements and early regression in Marinoan (635 Ma) cap dolostones: regional benchmarks of vanishing ice-sheets? *Earth Planet. Sci. Lett.* **300**, 374–384 (2010).

10 Zhou, G., Luo, T., Zhou, M., Xing, L. & Gan, T. A ubiquitous hydrothermal episode recorded in the sheet-crack cements of a Marinoan cap dolostone of South China: implication for the origin of the extremely ^{13}C-depleted calcite cement. *J. Asian Earth Sci.* **134**, 63–71 (2017).

11 Zhou, C., Bao, H., Peng, Y. & Yuan, X. Timing the deposition of ^{17}O-depleted barite at the aftermath of Nantuo glacial meltdown in South China. *Geology* **38**, 903–906 (2010).

12 Zhou, C., Huyskens, M. H., Lang, X., Xiao, S. & Yin, Q.-Z. Calibrating the terminations of Cryogenian global glaciations. *Geology* **47**, 251–254 (2019).

13 Condon, D. *et al.* U-Pb ages from the Neoproterozoic Doushantuo Formation, China. *Science* **308**, 95–98 (2005).

14 Barfod, G. H. *et al.* New Lu-Hf and Pb-Pb age constraints on the earliest animal fossils. *Earth Planet. Sci. Lett.* **201**, 203–212 (2002).

15 Liu, P., Yin, C., Gao, L., Tang, F. & Chen, S. New material of microfossils from the Ediacaran Doushantuo Formation in the Zhangcunping area, Yichang, Hubei Province and its zircon SHRIMP U-Pb age. *Chin. Sci. Bull.* **54**, 1058–1064 (2009).

16 Zhou, C. *et al.* A new SIMS zircon U–Pb date from the Ediacaran Doushantuo Formation: age constraint on the Weng'an biota. *Geol. Mag.* **154**, 1193–1201 (2017).

17 Corkeron, M. 'Cap carbonates' and Neoproterozoic glacigenic successions from the Kimberley region, north‐west Australia. *Sedimentology* **54**, 871‐903 (2007).

18 Zhao, Y.-Y., Zhao, M.-Y. & Li, S.-Z. Evidences of hydrothermal fluids recorded in microfacies of the Ediacaran cap dolostone: geochemical implications in South China. *Precambrian Res.* **306**, 1–21 (2018).

19 Sumrall, J., Mylroie, J. & Kambesis, P. Microbial mixing zone dolomitization and karst development within Isla de Mona Dolomite, Isla de Mona, Puerto Rico. *Carbonat. Evaporit.* **30**, 45–58 (2015).

20 Ren, M. & Jones, B. Genesis of island dolostones. *Sedimentology* **65**, 2003–2033 (2018).

21 Gan, T. *et al.* Miniature paleo-speleothems from the earliest Ediacaran (635 Ma) Doushantuo cap dolostone in South China and their implications for terrestrial ecosystems. *EarthArXiv*, https://doi.org/10.31223/osf.io/srkcp

(2019).
22 Borsato, A. *et al.* Holocene climate and environmental reconstruction from calcareous tufa and moonmilk deposits in Trentino caves. *Stud. Trent. Sci. Nat., Acta. Geol.* **82**, 239–259 (2005).
23 Baskar, S., Baskar, R. & Routh, J. Biogenic evidences of moonmilk deposition in the Mawmluh Cave, Meghalaya, India. *Geomicrobiol. J.* **28**, 252–265 (2011).
24 Cañaveras, J. C. *et al.* On the origin of fiber calcite crystals in moonmilk deposits. *Naturwissenschaften* **93**, 27–32 (2006).
25 Borsato, A., Frisia, S., Jones, B. & Van Der Borg, K. Calcite moonmilk: crystal morphology and environment of formation in caves in the Italian Alps. *J. Sediment. Res.* **70**, 1171–1182 (2000).
26 Lacelle, D., Lauriol, B. & Clark, I. D. Seasonal isotopic imprint in moonmilk from Caverne de l'Ours (Quebec, Canada): implications for climatic reconstruction. *Can. J. Earth Sci.* **41**, 1411–1423 (2004).
27 Cui, Y. *et al.* Germanium/silica ratio and rare earth element composition of silica-filling in sheet cracks of the Doushantuo cap carbonates, South China: constraining hydrothermal activity during the Marinoan snowball Earth glaciation. *Precambrian Res.* **332**, 105407 (2019).
28 Wang, J., Jiang, G., Xiao, S., Li, Q. & Wei, Q. Carbon isotope evidence for widespread methane seeps in the ca. 635 Ma Doushantuo cap carbonate in south China. *Geology* **36**, 347–350 (2008).
29 Wang, Z. *et al.* Raman geothermometry of carbonaceous material in the basal Ediacaran Doushantuo cap dolostone: the thermal history of extremely negative $\delta^{13}C$ signatures in the aftermath of the terminal Cryogenian snowball Earth glaciation. *Precambrian Res.* **298**, 174–186 (2017).
30 Lin, Z., Wang, Q., Feng, D., Liu, Q. & Chen, D. Post-depositional origin of highly ^{13}C-depleted carbonate in the Doushantuo cap dolostone in South China: insights from petrography and stable carbon isotopes. *Sediment. Geol.* **242**, 71–79 (2011).

31 Jiang, G., Kennedy, M. J. & Christie-Blick, N. Stable isotopic evidence for methane seeps in Neoproterozoic postglacial cap carbonates. *Nature* **426**, 822–826 (2003).

32 Bristow, T. F., Bonifacie, M., Derkowski, A., Eiler, J. M. & Grotzinger, J. P. A hydrothermal origin for isotopically anomalous cap dolostone cements from south China. *Nature* **474**, 68–71 (2011).

33 Tredici, S. M. *et al.* Calcite-forming *Bacillus licheniformis* thriving on underwater speleothems of a hydrothermal cave. *Geomicrobiol. J.* **35**, 804–817 (2018).

34 Barton, H. A., Spear, J. R. & Pace, N. R. Microbial life in the underworld: biogenicity in secondary mineral formations. *Geomicrobiol. J.* **18**, 359–368 (2001).

35 Axenov-Gibanov, D. V. *et al.* Actinobacteria isolated from an underground lake and moonmilk speleothem from the biggest conglomeratic karstic cave in Siberia as sources of novel biologically active compounds. *Plos One* **11**, e0149216 (2016).

36 Northup, D. *et al.* Biological investigations in Lechuguilla Cave. *NSS Bulletin* **56**, 54–63 (1994).

37 Nieves-Rivera, Á. M., Santos-Flores, C. J., Dugan, F. M. & Miller, T. E. Guanophilic fungi in three caves of southwestern Puerto Rico. *Int. J. Speleol.* **38**, 61–70 (2009).

38 Nov ákov á, A. Microscopic fungi isolated from the Domica Cave system (Slovak Karst National Park, Slovakia). A review. *Int. J. Speleol.* **38**, 71–82 (2009).

39 Popović, S. *et al.* Cyanobacteria, algae and microfungi present in biofilm from Božana Cave (Serbia). *Int. J. Speleol.* **44**, 141–149 (2015).

40 Joanna, C.-M. & Andrzej, M. in *Cyanobacteria* (ed Tiwari, A.) 137–164 (InTech, 2018).

41 Czerwik-Marcinkowska, J., Wojciechowska, A. & Massalski, A. Biodiversity of limestone caves: aggregations of aerophytic algae and cyanobacteria in

relation to site factors. *Polish J. Ecol.* **63**, 481–499 (2015).

42 Baskar, S. *et al.* Evidences for microbial precipitation of calcite in speleothems from Krem Syndai in Jaintia Hills, Meghalaya, India. *Geomicrobiol. J.* **33**, 906–933 (2016).

43 Kondratyeva, L. M., Polevskaya, O. S., Litvinenko, Z. N., Golubeva, E. M. & Konovalova, N. S. Role of the microbial community in formation of speleothem (moonmilk) in the Snezhnaya carst cave (abkhazia). *Mikrobiologiia* **85**, 598–608 (2016).

44 Tisato, N. *et al.* Microbial mediation of complex subterranean mineral structures. *Sci. Rep.* **5**, 15525 (2015).

45 Fairchild, I. J. & Baker, A. *Speleothem Science: From Process to Past Environments*. (Wiley-Blackwell, 2012).

46 de Cisneros, C. J., Molina, J. M., Nieto, L. M., Ruiz-Ortiz, P. A. & Vera, J. A. Calcretes from a palaeosinkhole in Jurassic palaeokarst (Subbetic, southern Spain). *Sediment. Geol.* **87**, 13–24 (1993).

47 Amodio, S., Barattolo, F. & Riding, R. Early Cretaceous dendritic shrub-like fabric in karstified peritidal carbonates from southern Italy. *Sediment. Geol.* **373**, 134–146 (2018).

48 Grimes, K. G. The water below: an introduction to karst hydrology and the hydrological setting of the Australian karsts. *Proceedings of the 13th Australasian Conference on Cave and Karst Management*, 24–31 (1999).

49 Zhang, J. *et al.* Improved precision and spatial resolution of sulfur isotope analysis using NanoSIMS. *J. Anal. At. Spectrom.* **20**, 1934–1943 (2014).

50 Chen, L. *et al.* Extreme variation of sulfur isotopic compositions in pyrite from the Qiuling sediment-hosted gold deposit, West Qinling orogen, central China: an in situ SIMS study with implications for the source of sulfur. *Mineral. Deposita* **50**, 643–656 (2015).
