# Supplementary material for: Cryptic terrestrial fungus-like fossils of the early Ediacaran Period
Source: Nat Commun. 2021 Jan 28;12:641. doi: 10.1038/s41467-021-20975-1 (PMC7843733; doi:10.1038/s41467-021-20975-1)
Supplement: Supplementary file 3 — Description of Additional Supplementary Files [file 41467_2021_20975_MOESM3_ESM.pdf]

Description of Additional Supplementary Files

File Name: Supplementary Movie 1
Description: CLSM video corresponding to CLSM micrograph in Figure 2h.

File Name: Supplementary Movie 2
Description: SRXTM video corresponding to SRXTM surface renderings in Figure 4g.
