# Supplementary material for: Cryptic terrestrial fungus-like fossils of the early Ediacaran Period
Source: Nat Commun. 2021 Jan 28;12:641. doi: 10.1038/s41467-021-20975-1 (PMC7843733; doi:10.1038/s41467-021-20975-1)
Supplement: Supplementary file 6 — Reporting Summary [file 41467_2021_20975_MOESM6_ESM.pdf]

nature research

Corresponding author(s): Ke Pang

Last updated by author(s): Dec 14, 2020

Reporting Summary

Nature Research wishes to improve the reproducibility of the work that we publish. This form provides structure for consistency and transparency in reporting. For further information on Nature Research policies, see our Editorial Policies and the Editorial Policy Checklist.

Statistics

For all statistical analyses, confirm that the following items are present in the figure legend, table legend, main text, or Methods section.

n/a	Confirmed	
☐	☒	The exact sample size (*n*) for each experimental group/condition, given as a discrete number and unit of measurement
☐	☒	A statement on whether measurements were taken from distinct samples or whether the same sample was measured repeatedly
☒	☐	The statistical test(s) used AND whether they are one- or two-sided<br>*Only common tests should be described solely by name; describe more complex techniques in the Methods section.*
☒	☐	A description of all covariates tested
☒	☐	A description of any assumptions or corrections, such as tests of normality and adjustment for multiple comparisons
☐	☒	A full description of the statistical parameters including central tendency (e.g. means) or other basic estimates (e.g. regression coefficient) AND variation (e.g. standard deviation) or associated estimates of uncertainty (e.g. confidence intervals)
☒	☐	For null hypothesis testing, the test statistic (e.g. *F*, *t*, *r*) with confidence intervals, effect sizes, degrees of freedom and *P* value noted<br>*Give P values as exact values whenever suitable.*
☒	☐	For Bayesian analysis, information on the choice of priors and Markov chain Monte Carlo settings
☒	☐	For hierarchical and complex designs, identification of the appropriate level for tests and full reporting of outcomes
☒	☐	Estimates of effect sizes (e.g. Cohen's *d*, Pearson's *r*), indicating how they were calculated

Our web collection on statistics for biologists contains articles on many of the points above.

Software and code

Policy information about availability of computer code

Data collection	PITRE V3.1 software; ImageJ (1.47v); FV10-ASW (v.3.01)
Data analysis	VG Studio 2.0; Microsoft Excel 2013; OMNIC 8.0 software; Isoplot 3.0; ICPMSDataCal (v.11); LabSpec (v.5)

For manuscripts utilizing custom algorithms or software that are central to the research but not yet described in published literature, software must be made available to editors and reviewers. We strongly encourage code deposition in a community repository (e.g. GitHub). See the Nature Research guidelines for submitting code & software for further information.

Data

Policy information about availability of data

All manuscripts must include a data availability statement. This statement should provide the following information, where applicable:
- Accession codes, unique identifiers, or web links for publicly available datasets
- A list of figures that have associated raw data
- A description of any restrictions on data availability

The data that support the findings of this study are available in the paper and its Supplementary Information, or from the corresponding authors upon reasonable request. All specimens illustrated in this paper are reposited in Nanjing Institute of Geology and Palaeontology (NIGPAS, Nanjing, China), with NIGPAS museum catalog numbers (prefix PB-) given for illustrated specimens (see Supplementary Table 3 for repository information). The source data underlying Supplementary Figures 7 and 8 is provided in Supplementary Tables 1 and 2.

Field-specific reporting

Please select the one below that is the best fit for your research. If you are not sure, read the appropriate sections before making your selection.

☐ Life sciences ☐ Behavioural & social sciences ☒ Ecological, evolutionary & environmental sciences

For a reference copy of the document with all sections, see nature.com/documents/nr-reporting-summary-flat.pdf

Ecological, evolutionary & environmental sciences study design

All studies must disclose on these points even when the disclosure is negative.

Study description	Palaeontological study of pyritized fungus-like microfossils preserved in siliceous cements of sheet-cavities from the basal Ediacaran Doushantuo Formation (~635 Ma) at Weng'an, South China. Total number of measured specimens was 199. Measurement were repeated 3 times.
Research sample	Nine sheet-cavity samples (16DT-2, 16DT-2b, 16DTC-2A ,17DT-A-1, 17DT-A-2, 17DT-A-4, 17DT-A-6, 17DT-A-9, and 17DT-2A) were collected from the cap dolostone of the Doushantuo Formation at the Datang section (27°01′54.5″N, 107°24′08.4″E) and eleven sheet-cavity samples (18BD-6, 18BD-8, 18BD-9-1, 18BD-12, 18BD-20b, 18BD-25, 18BD-33, 18BD-32, 18BD-34, 18BD-35, and 18BD-36) were collected from the cap dolostone of the Doushantuo Formation at the Beidoushan section (27°01′40.4″N, 107°23′ 22.0″E), Weng'an, Guizhou Province, South China. All specimens illustrated in this paper are reposited in Nanjing Institute of Geology and Palaeontology (NIGPAS, Nanjing, China), with NIGPAS museum catalog numbers (prefix PB-) given for illustrated specimens (see Supplementary Table 3 for repository information).
Sampling strategy	All specimens encountered in the petrographic thin sections were studied. A total of 119 Type A microfossil specimens and 80 Type B microfossil specimens were measured. This sample size is deemed sufficient in paleontological investigations. No sample size calculation was performed.
Data collection	Microfossil specimens were photographed on a Leica DM4500P microscope coupled with a Nikon D750 digital camera at the Institute of Geochemistry, Chinese Academy of Sciences, a Zeiss Axioscope A1 microscope with a Axiocam 506 digital camera at the Nanjing Institute of Geology and Palaeontology, Chinese Academy of Sciences, a Zeiss Axioscope A1 microscope with a Axiocam 512 digital camera at Virginia Tech, an Olympus SZ16 microscope with a DP27 digital camera at Virginia Tech, and an Olympus BX-60 upright microscope with an Olympus SC30 digital camera at the University of Cincinnati. Measurements were made on photographs of specimens using ImageJ and recorded by Tian Gan.
Timing and spatial scale	Sheet-cavity samples were collected in 2016-2018 from the cap dolostone of the Doushantuo Formation at the Datang section (27° 01′54.5″N, 107°24′08.4″E) and at the Beidoushan section (27°01′40.4″N, 107°23′22.0″E), Weng'an, Guizhou Province, South China.
Data exclusions	No data were excluded from analysis.
Reproducibility	Each specimens was measured 3 times and all attempts at replication were successful. To ensure reproducibility, details about fossil locality and stratigraphic horizon have been noted.
Randomization	Our study did not require randomization because it did not involve experiments.
Blinding	Our study did not require blinding because it did not involve experiments.

Did the study involve field work? ☒ Yes ☐ No

Field work, collection and transport

Field conditions	The field site is located in the subtropical zone. Climate is cool in the field season (autumn time). Outcrops of cap dolostone are well exposed.
Location	Located at the Datang section (27°01′54.5″N, 107°24′08.4″E) and at the Beidoushan section (27°01′40.4″N, 107°23′22.0″E), Weng'an, Guizhou Province, South China
Access & import/export	Collection of fossil specimens was carried out in a responsible manner and in compliance with the local, national and international laws. All specimens illustrated in this paper are reposited in Nanjing Institute of Geology and Palaeontology (NIGPAS, Nanjing, China), with NIGPAS museum catalog numbers (prefix PB-) given for illustrated specimens (see Supplementary Table 3 for repository information).
Disturbance	We collect 20 palm-sized sheet-cravity samples and are pretty sure that disturbance was minimized.

Reporting for specific materials, systems and methods

We require information from authors about some types of materials, experimental systems and methods used in many studies. Here, indicate whether each material, system or method listed is relevant to your study. If you are not sure if a list item applies to your research, read the appropriate section before selecting a response.

Materials & experimental systems

n/a	Involved in the study
☒	☐ Antibodies
☒	☐ Eukaryotic cell lines
☐	☒ Palaeontology and archaeology
☒	☐ Animals and other organisms
☒	☐ Human research participants
☒	☐ Clinical data
☒	☐ Dual use research of concern

Methods

n/a	Involved in the study
☒	☐ ChIP-seq
☒	☐ Flow cytometry
☒	☐ MRI-based neuroimaging

Palaeontology and Archaeology

Specimen provenance

Cap dolostone of the Doushantuo Formation, at the Datang section (27°01′54.5″N, 107°24′08.4″E) and at the Beidoushan section (27°01′40.4″N, 107°23′22.0″E), Weng'an, Guizhou Province, South China. Collection permits were not needed.

Specimen deposition

All specimens illustrated in this paper are reposited in Nanjing Institute of Geology and Palaeontology (NIGPAS, Nanjing, China), with NIGPAS museum catalog numbers (prefix PB-) given for illustrated specimens (see Supplementary Table 3 for repository information).

Dating methods

The age of the Doushantuo cap dolostone is well constrained to be ca. 635 Ma by a published U-Pb zircon age of 634.6 ± 0.9 Ma from the topmost Nantuo Formation and a published U-Pb zircon age of 635.2 ± 0.6 Ma from the top of cap dolostone in South China (Extended Data Fig. 1c, d). A new in-situ LA-ICP-MS U-Pb age of 632 ± 17 Ma is obtained from the isopachous dolomite cement in Doushantuo sheet-cavities in this study. U-Pb age of 632 ± 17 Ma using LA-ICP-MS, including the standards NIST 614 and WC-1 for normalization of 207Pb/206Pb and 238U /206Pb ratios, was described previously in refs.70,71. U-Pb isotopes were measured in-situ on isopachous dolomite (ID) in sheet-cavities from the Daping section (Hunan Province of South China) on an Agilent 7900 quadruple mass spectrometer equipped with an inductively coupled plasma and a GeoLasPro 193 nm ArF excimer laser (LA-ICP-MS) at the State Key Laboratory of Ore Deposit Geochemistry, Institute of Geochemistry, Chinese Academy of Sciences. Samples were ablated by a laser of 120 µm spot size, 10 Hz repetition, and 8 J•cm−2 energy density. The aerosols were carried by pure Helium (450 ml/min) and then mixed with argon via a T-connector and finally injected to the ICP torch. Pure nitrogen (~3.0 ml/min) was added to the helium carrier gas to increase the sensitivity via a Y-junction before entering ICP (ref.72). Samples were pre-ablated for 3-5 pulses before analysis to remove surface contaminations. Each analysis included 20 seconds for background acquisition, 30 seconds for data acquisition, and 40 seconds for elimination of memory effects. The approach of standard-sample bracketing was used to measure the isotopes of 202, 204, 206, 207, 208, 232, and 238. Data reduction was undertaken off-line using ICPMSDataCal (ref.73) and then regressed to determine a discordia line on Tera-Wasserburg model using Isoplot 3.0. U-Pb isotopic data are presented in Supplementary Figure 8 and Supplementary Table 2.

☒ Tick this box to confirm that the raw and calibrated dates are available in the paper or in Supplementary Information.

Ethics oversight

No ethical approval or guidance was required for this palaeontological study.

Note that full information on the approval of the study protocol must also be provided in the manuscript.
